# Carotid characteristics of black South Africans with five-year sustained hypertension

**DOI:** 10.5830/CVJA-2016-059

**Published:** 2016

**Authors:** Melissa Maritz, Carla MT Fourie, Johannes M van Rooyen, Hugo W Huisman, Aletta E Schutte, Aletta E Schutte

**Affiliations:** Hypertension in Africa Research Team (HART ), North-West University, Potchefstroom, South Africa; Hypertension in Africa Research Team (HART ), North-West University, Potchefstroom, South Africa; Hypertension in Africa Research Team (HART ), North-West University, Potchefstroom, South Africa; Hypertension in Africa Research Team (HART ), North-West University, Potchefstroom, South Africa; Hypertension in Africa Research Team (HART ), North-West University, Potchefstroom, South Africa; MRC Research Unit for Hypertension and Cardiovascular Disease, North-West University, Potchefstroom, South Africa

**Keywords:** ethnicity, large artery, stiffness, distensibility, hypertension, central pressure

## Abstract

**Introduction:**

An important feature of hypertension is a reduction in large artery distensibility, which may be due to structural and functional adaptations. Black populations are particularly prone to the development of hypertension. We therefore compared the carotid characteristics between fiveyear sustained hypertensive and normotensive black South Africans, and investigated how carotid characteristics relate to cardiometabolic risk factors, inflammation, endothelial activation and health behaviours.

**Methods:**

We included HIV-free black South Africans who were either consistently hypertensive (n = 351) or normotensive (n = 241) from 2005 to 2010. We assessed carotid characteristics, including intima–media thickness (IMT), distensibility and lumen diameter with B-mode ultrasound, and calculated Young’s elastic modulus, cross-sectional wall area and beta-stiffness index. We measured the carotid dorsalis pedis pulse-wave velocity, brachial and central systolic blood pressure (cSBP) and determined metabolic, inflammatory and endothelial activation markers from blood samples. Health behaviours were reported in questionnaires.

**Results:**

The hypertensive group presented with higher brachial and central blood pressure, thicker IMT and stiffer carotid arteries (all p < 0.001). However, after adjustment for cSBP but not mean arterial pressure (MAP), all significant differences in carotid characteristics were lost. The carotid thickness measurements did not differ after adjustment for MAP. After adjustment, metabolic, inflammatory and endothelial activation markers did not differ between the two groups.

**Conclusion:**

Our results suggest that besides structural changes, functional adaptations are also involved in deterioration of the carotid wall characteristics of hypertensive black South Africans. These results highlight the importance of proper hypertension control in Africa.

## Introduction

Worldwide, cardiovascular disease is the leading cause of death.^1^ Arterial stiffness is implicated in the development of cardiovascular disease, which results in stroke, coronary heart disease and heart failure.^2^,^3^ Arterial stiffness refers to the reduced ability of an artery to expand and recoil with pressure changes, and the arterial distensibility is a measure of vessel stiffness.^4^ Decreased distensibility may raise central systolic blood pressure (cSBP) and consequently decrease the amplification of pulse pressure.^5^,^6^ This results in inadequate coronary perfusion, increased afterload on the heart, as well as an increased pulsatile load on the microcirculation.^3^

The artery is able to resist strain during blood pressure increases via recruitment of collagen fibres in the arterial wall,^7^ but sustained high pressure predisposes the vessel to progressive changes in the wall shape and composition, ultimately leading to several clinical complications such as arterial fibrosis and stiffening.^8^,^9^

Recently, van Sloten et al. reported that in a European population, carotid artery stiffness independently predicts incident cardiovascular (CV) events and all-cause mortality.^3^ The higher incidence of stroke, heart failure and renal failure in black populations are a consequence of the higher prevalence of hypertension,^10^,^11^ and arterial stiffness^12^,^13^ in black compared to white populations.

Despite the high prevalence of hypertension and stroke among black South Africans,^14^ there is limited knowledge on carotid wall properties in this population. We therefore compared the characteristics of the carotid arteries between normotensive and five-year sustained hypertensive black individuals, along with brachial and central blood pressure and conventional cardiometabolic risk factors, markers of inflammation, endothelial activation and health behaviours.

## Methods

**Fig. 1:**
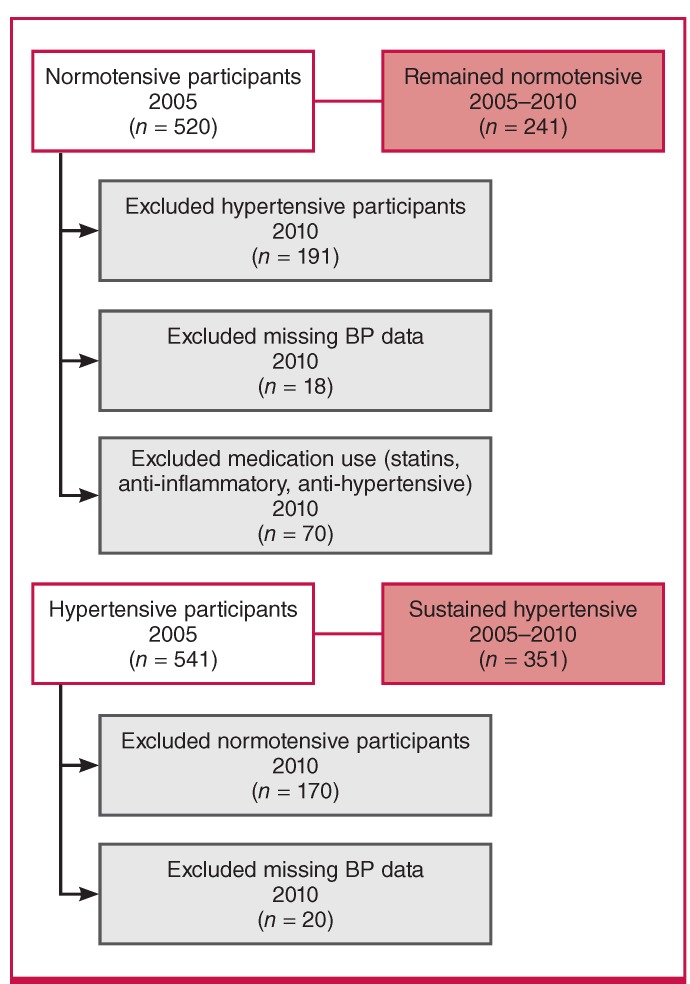
Study population; all participants were HIV-free. BP, blood pressure.

This sub-study forms part of the South African leg of the multi-national Prospective Urban and Rural Epidemiology (PURE) study.^15^ The participants of the PURE-SA study were from urban and rural localities in the North West Province,and baseline data collection took place in 2005 (n = 2 010 participants), while follow-up data was collected five years later, in 2010 (n = 1 288 participants). For this sub-study we included only the HIV-free black participants with two consecutive (2005, 2010) blood pressure measurements in either the hypertensive or normotensive range (n = 592 participants), consisting of a group of five-year sustained normotensive (n = 241participants) and hypertensive (n = 351 participants) black South Africans (Fig. 1).

The Health Research Ethics Committee of the North-West University approved the protocol of the PURE-SA study, as well as this sub-study (ethics number: NWU-00016-10-A1), and it complies with the Declaration of Helsinki. Participants completed structured demographic, lifestyle and physical activity questionnaires with the assistance of trained African field workers from the communities. Before measurements commenced, all procedures were explained to the participants in their home language. The participants then gave written informed consent.

Height (Invicta stadiometer IP 1465, Leicester, UK), weight (Precision health scale, A & D Company, Tokyo, Japan) and waist circumference (WC) (Holtain unstretchable metal tape, Apex Tool Group, Apex, USA) were measured using standardised methods and calibrated instruments.^16^ Body mass index (BMI) was calculated with the formula: weight (kg)/height (m^2^).

The validated OMRON HEM-757 (Omron Healthcare, Kyoto, Japan) device was used to measure blood pressure. Each participant was fitted with the correct cuff size. During the measurements, the participant was seated in a relaxed upright position with legs uncrossed. After a resting period of 10 minutes, the brachial systolic (bSBP) and diastolic blood pressure (bDBP) were measured on the right upper arm, followed by a five-minute resting period and a second blood pressure measurement. The value of the second measurement was used for analysis. Participants were classified as hypertensive or normotensive according to standard guidelines.^17^

The cSBP was measured with the OMRON 9000AI device (Omron Healthcare, Kyoto, Japan), which uses the second systolic peak (reflected wave) as basis for the calculation of cSBP. Diastolic blood pressure is assumed to be consistent throughout the body, therefore the central pulse pressure (cPP) was calculated by subtracting the bDBP from the cSBP.^18^,^19^

Pulse-wave velocity (PWV) was measured non-invasively on the left side of each participant while in a supine position. The Complior SP device (Artech-Medical, Pantin, France) uses superficial pulses over the carotid dorsalis pedis (cdPWV) section of the arterial tree to estimate PWV.

The carotid characteristics were measured non-invasively using B-mode ultrasonography with the SonoSite Micromaxx system (SonoSite, Inc., Bothel, WA, USA), using a six- to 13-MHz linear array transducer. A minimum of two optimal angles were used from images of the right and left common carotid arteries. These images were then measured by a single reader according to protocols, and digitised and imported into the automated software of the Artery Measurements System (Gothenburg, Sweden).

The carotid intima–media thickness (IMT) was analysed by a single reader on a good-quality image of a maximum 10-mm segment. The borders of the inner diameter of the blood vessel and the near and far wall of the intima–media were identified by an automated function of the program, but user intervention was possible. Approximately 100 discrete measurement points within a 10-mm segment of the carotid artery were used to obtain the mean IMT and the carotid diameter for each participant.

The carotid cross-sectional wall area (CSWA) was calculated according to the following formula: CSWA = [3.14 × ( LD/2 + IMTf) × ( LD/2 + IMTf)] – [3.14 × (LD/2) × (LD/2) ] where LD = lumen diameter and IMTf = carotid intima–media thickness of the far wall.

A single reader analysed video-clips of the carotid artery in order to determine the maximum and minimum lumen diameters (LD). The carotid distensibility (CD) coefficient was calculated according to the following formula: CD = [(2 × delta LD × min LD) + (delta LD)^2^)/(cPP × min LD^2^] where LD = lumen diameter, delta LD = maximum LD – minimum LD, min LD = minimum LD, cPP = central pulse pressure.^20^

Young’s elastic modulus was calculated according to the following formula: Young’s elastic modulus = min LD/(IMT × distensibility) where IMT = carotid intima–media thickness, min LD = minimum lumen diameter.^21^

The beta-stiffness index was calculated according to the following formula: Beta-stiffness index = ln (cSBP/dDBP) / deltaLD/min LD where delta LD = maximum LD – minimum LD, min LD = minimum LD.^22^

Participants were required to fast for at least eight hours and blood samples were taken from the ante-brachial vein with a sterile winged infusion set and syringes. The preparation of the serum and plasma was done according to standardised methods, snap frozen on dry ice and stored in the laboratory at –80°C. In the case of blood collection in a rural area, serum and plasma were snap frozen and stored at –20°C for not more than five days. The serum was then transported to the laboratory and stored at –80°C for further analysis.

The Cobas Integra 400 (Roche® Clinical System, Roche Diagnostics, Indianapolis, IN) was used to assess the quantitative aspect of total cholesterol (TC), high-density lipoprotein cholesterol (HDL-C), triglycerides (TG), gamma-glutamyltransferase (GGT), creatinine and high-sensitivity C-reactive protein (hsCRP) levels in the serum samples, and glucose levels in fluoride plasma samples. Glycosylated haemoglobin (HbA_1c_) levels were determined from EDTA whole blood with ion-exchange high-performance liquid chromatography (D-10 haemoglobin testing system, Bio-Rad #220-0101).

The Friedewald formula was used to calculate the quantitative aspect of low-density lipoprotein cholesterol (LDL-C).^23^ The estimated creatinine clearance (CrCl) rate was calculated with the Cockcroft–Gault formula.^24^

We determined serum interleukin-6 (IL-6) levels with an electro-chemiluminescence immunoassay (Cobas e411 analyzer,Roche, Basel, Switzerland). Serum intercellular adhesion molecule 1 (sICAM-1) and vascular cell adhesion molecule 1 (sVCAM- 1) concentrations were assessed by sandwich ELISAs (human sICAM-1 and human sVCAM-1 assay, IBL, Hamburg, Germany).

## Statistical analysis

Statistical analyses were performed using Statistica® 12 (StatSoft, Inc, Tulsa, OK, USA). Descriptive statistics, including the mean and standard deviation, were performed on data with a normal distribution. If not normally distributed, the data were logarithmically transformed and presented as the geometric mean and the fifth and 95th percentiles.

We used independent t-tests to determine differences between normotensives and hypertensives or chi-squared tests for categorical variables. We compared the groups using ANCOVA while adjusting for age, gender, WC, GGT, tobacco and antihypertensive medication use. We plotted quartiles of cSBP against carotid features, and compared carotid features using ANOVA and ANCOVA while adjusting for cSBP

We used single and linear regression analyses to determine associations between carotid measures and cardiometabolic risk factors, health behaviours, inflammation and endothelial activation. Multiple regression analyses were done to determine independent associations between the carotid characteristics and cardiometabolic risk factors, with the dependent variables including CD and IMT, and CSWA and max LD. The following co-variates were included in the regression model: locality, gender, age, WC, cSBP, heart rate, LDL-C, HbA_1c_, CrCl, hsCRP, ICAM-1, GGT, tobacco use and additionally for the hypertensive group, anti-hypertensive medication use.

## Results

The characteristics of normotensive and hypertensive black Africans are shown in Table 1. The hypertensives were older, had a higher BMI and WC, and a larger percentage were from an urban area than the normotensives. All the cardiovascular measurements, as well as carotid characteristics, glycaemic and inflammatory markers, ICAM-1, TG, GGT and self-reported alcohol use were significantly higher in the hypertensive group.

**Table 1:** Comparison of case group with control group

	*Normotensive (n = 241)*	*Hypertensive (n = 351)*	*p-value*
Men, n (%)	90 (37.3)	129 (36.8)	0.88
Urban, n (%)	78 (32.4)	178 (50.7)	< 0.001
Age, years	52.8 ± 8.93	59.0 ± 10.0	< 0.001
Anthropometry			
Waist circumference, cm	78.7 ± 11.9	84.7 ± 13.5	< 0.001
Body mass index, kg/m^2^	24.7 ± 6.87	26.6 ± 7.85	0.003
Cardiovascular measures			
Brachial SBP, mm Hg	119 ± 11.9	157 ± 22.4	< 0.001
Brachial DBP, mm Hg	78.9 ± 7.23	100 ± 12.5	< 0.001
Heart rate, bpm	61.8 ± 14.7	67.8 ± 17.9	< 0.001
Central SBP, mm Hg	116 ± 12.8	150 ± 22.2	< 0.001
Central PP, mm Hg	37.4 ± 11.8	50.9 ± 20.4	< 0.001
Carotid dorsalis pedis PWV, m/s	8.25 ± 1.35	10.0 ± 1.89	< 0.001
Carotid characteristics			
Distensibility × 10^-3^, 1/kPa	4.72 ± 2.00	3.02 ± 1.83	< 0.001
Young’s elastic modulus × 10^3^, kPa	2.17 ± 1.07	3.72 ± 2.20	< 0.001
Beta-stiffness index	7.10 ± 2.62	9.37 ± 4.51	< 0.001
Intima–media thickness, mm	0.68 ± 0.13	0.77 ± 0.17	< 0.001
Cross-sectional wall area, mm^2^	14.2 ± 4.41	17.4 ± 5.30	< 0.001
Lumen diameter maximum, mm	6.18 ± 0.77	6.60 ± 0.85	< 0.001
Lumen diameter minimum, mm	5.74 ± 0.72	6.22 ± 0.83	< 0.001
Lipids			
HDL-C, mmol/l	1.45 ± 0.65	1.51 ± 0.60	0.30
LDL-C, mmol/l	2.86 ± 1.17	2.96 ± 1.14	2.96 ± 1.14 0.29
Triglycerides, mmol/l	1.09 (0.57–2.11)	1.20 (0.59–2.89)	0.039
Glycaemia			
Glucose, mmol/l	4.86 (3.91–6.06)	5.27 (3.96–7.90)	< 0.001
HbA_1c_ (%)	5.94 (5.30–6.80)	6.08 (5.20–7.80)	0.038
Inflammatory markers			
Interleukin-6, pg/ml	3.39 (0.75–23.5)	4.43 (0.75–20.0)	< 0.001
C-reactive protein, mg/l	2.96 (0.21–32.6)	3.84 (0.38–28.4)	0.022
Adhesion molecules			
Intercellular adhesion molecule-1, pg/ml	286 ± 92.7	321 ± 111	< 0.001
Vascular adhesion molecule-1, pg/ml	732 (442–1388)	762 (443–1735)	0.23
Renal function			
Creatinine clearance, ml/min	91.4 (53.7–163)	91.6 (47.7–164)	0.94
Health behaviours			
γ-glutamyl transferase, U/l	33.7 (11.9–167)	52.7 (13.6–347)	< 0.001
Self-reported alcohol intake, n, total (%)	60/220 (27.3)	159/336 (47.3)	< 0.001
Self-reported tobacco use, n, total (%)	117/229 (51.1)	159/341 (46.6)	0.56
Anti-hypertension medication, n, total (%)	-	124/351 (35.3)	
Lipid-lowering medication, n, total (%)	-	5/351 (1.42)	
Anti-inflammatory medication, n, total (%)	-	21/351 (5.98)	

Data are arithmetic means ± SD or geometric mean (fifth and 95th percentile intervals) for logarithmically transformed.

n, number of participants; SBP, systolic blood pressure; DBP, diastolic blood pressure; PP, pulse pressure; PWV, pulse wave velocity; HDL-C, high-density lipoprotein; LDL-C, low-density lipoprotein; HbA1c, glycated haemoglobin.

After adjustments for age, gender, WC, GGT, tobacco and anti-hypertensive medication use (Table 2), all carotid characteristics except IMT remained significantly different. The inflammatory and glycaemic markers, lipids and adhesion molecules did not differ however after the above adjustments.

**Table 2:** Adjusted characteristics of normotensive and hypertensive black Africans

	*Normotensive (n = 241)*	*Hypertensive (n = 351)*	*p-value*
Cardiovascular measures			
Brachial SBP, mm Hg	120 ± 20.0	155 ± 20.7	< 0.001
Brachial DBP, mm Hg	79.2 ± 11.1	99.8 ± 11.6	< 0.001
Heart rate, bpm	62.7 ± 17.5	66.8 ± 18.0	0.017
Central SBP, mm Hg	117 ± 20.2	148 ± 20.9	< 0.001
Central PP, mm Hg	38.4 ± 17.9	49.4 ± 18.5	< 0.001
Carotid dorsalis pedis PWV, m/s	8.87 ± 2.06	9.37 ± 2.13	< 0.001
Carotid characteristics			
Distensibility × 10^-3^, 1/kPa	4.58 ± 1.98	3.15 ± 2.07	< 0.001
Young’s elastic modulus × 10^3^, kPa	2.26 ± 1.98	3.59 ± 2.07	< 0.001
Beta-stiffness index	7.48 ± 3.98	8.97 ± 4.16	0.002
Intima–media thickness, mm	0.72 ± 0.14	0.73 ± 0.18	0.42
Cross-sectional wall area, mm^2^	14.8 ± 4.71	16.7 ± 4.86	< 0.001
Lumen diameter maximum, mm	6.24 ± 0.85	6.51 ± 0.92	0.005
Lumen diameter minimum, mm	5.80 ± 0.85	6.12 ± 0.76	< 0.001
Lipids			
HDL-C, mmol/l	1.46 ± 0.60	1.50 ± 0.73	0.47
LDL-C, mmol/l	2.93 ± 1.19	2.90 ± 1.27	0.81
Triglycerides, mmol/l	1.16 (1.08–1.24)	1.11 (1.05–1.19)	0.50
Glycaemia			
Glucose, mmol/l	5.01 (4.86–5.16)	5.10 (4.97–5.23)	0.43
HbA^1c^, %	6.05 (5.93–6.15)	5.99 (5.89–6.08)	0.44
Inflammatory markers			
Interleukin-6, pg/ml	3.62 (3.19–4.11)	4.05 (3.64–4.51)	0.22
C-reactive protein, mg/l	3.41 (2.84–4.08)	3.18 (2.72–3.72)	0.62
Adhesion molecules			
Intracellular adhesion molecule-1, pg/ml	293 ± 101	308 ± 105	0.13
Vascular adhesion molecule-1, pg/ml	748 (706–792)	747 (711–785)	0.98
Creatinine clearance, ml/min	90.8 (87.8–94.0)	92.3 (89.6–95.0)	0.54

Data are arithmetic means ± SD or geometric mean (fifth and 95th percentile intervals) for logarithmically transformed. Data are adjusted for age, gender, waist circumference, γ-glutamyl transferase, tobacco use and anti-hypertensive medication use. Pulse-wave velocity and carotid intima–media thickness additionally adjusted for mean arterial pressure.

n, number of participants; SBP, systolic blood pressure; DBP, diastolic blood pressure; PP, pulse pressure; HDL-C, high-density lipoprotein cholesterol; LDL-C, low-density lipoprotein cholesterol; HbA_1c_, glycated haemoglobin.

**Fig. 2:**
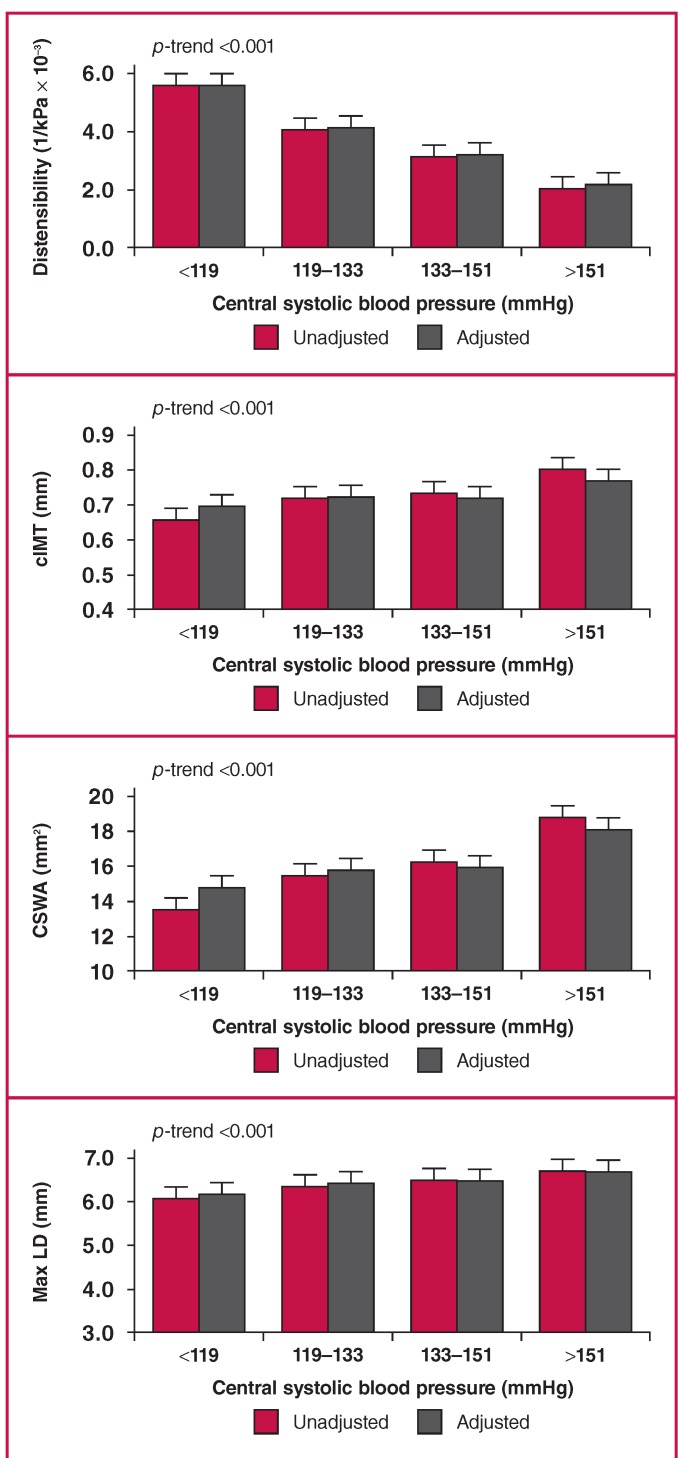
Quartiles of central blood pressure plotted against measures of the carotid artery in the total group (n = 592), unadjusted and adjusted for age, gender, waist circumference, GGT, tobacco and anti-hypertensive medication use. cIMT, carotid intima–media thickness; CSWA, cross-sectional wall area; Max LD, maximum lumen diameter.

The carotid characteristics plotted against quartiles of cSBP are shown in Fig. 2. All the carotid characteristics changed significantly with increasing cSBP before and after the adjustments.

We finally adjusted for cSBP when comparing the normotensive and hypertensive groups (Table 3), resulting in no significant differences in the carotid characteristics between the groups. When cSBP was substituted with either brachial SBP (p = 0.029) or mean arterial pressure (MAP) (p = 0.0012), the difference in distensibility remained, but the more physical measures such as IMT, CSWA and LD did not differ.

**Table 3:** Carotid characteristics of normotensive and hypertensive black Africans, adjusted for potential confounders

	*Normotensive (n = 241)*	*Hypertensive (n = 351)*	*p-value*
Carotid characteristics after adjustment for central SBP			
Distensibility × 10^-3^, 1/kPa	3.77 ± 1.98	3.95 ± 2.22	0.50
Young’s elastic modulus × 10^3^, kPa	3.04 ± 1.84	2.80 ± 2.05	0.34
Beta-stiffness index	8.43 ± 4.21	8.02 ± 4.61	0.47
Intima–media thickness, mm	0.71 ± 0.14	0.73 ± 0.17	0.19
Cross-sectional wall area, mm^2^	15.2 ± 4.96	16.2 ± 5.25	0.09
Lumen diameter maximum, mm	6.30 ± 0.82	6.41 ± 1.04	0.34
Lumen diameter minimum, mm	5.85 ± 0.94	6.02 ± 0.89	0.12
Carotid characteristics after adjustment for mean arterial pressure			
Distensibility × 10^-3^, 1/kPa	4.38 ± 2.45	3.35 ± 2.66	0.0034
Young’s elastic modulus × 10^3^, kPa	2.46 ± 2.30	3.39 ± 2.64	0.0082
Beta-stiffness index	7.13 ± 4.68	9.32 ± 5.20	< 0.001
Intima–media thickness, mm	0.71 ± 0.14	0.73 ± 0.18	0.42
Cross-sectional wall area, mm^2^	15.6 ± 5.57	16.0 ± 6.07	0.35
Lumen diameter maximum, mm	6.30 ± 0.97	6.44 ± 1.07	0.32
Lumen diameter minimum, mm	5.86 ± 0.97	6.06 ± 0.92	0.17

Data are arithmetic means ± SD. Data adjusted for age, gender, waist circumference, γ-glutamyl transferase, tobacco and anti-hypertensive medication use.

In sensitivity analysis, we further compared hypertensives who were not using anti-hypertensive therapy (n = 227) with treated hypertensives (n = 124) and normotensives, applying similar adjustments, including cSBP. We found results similar to those in Table 3 (Table 4). Furthermore, excluding participants on antihypertensive medication and comparing the normotensive group to the treated hypertensive group did not change the results. The types of medication used by the hypertensive participants are shown in Table 5.

**Table 4:** Comparison of case group with control group

*Carotid characteristics*	*Normotensive (n = 241)*	*Untreated hypertensives (n = 227)*	*Treated hypertensives (n = 124)*	*p-value*
Distensibility × 10^-3^, 1/kPa	3.53 ± 2.10	3.70 ± 1.77	3.47 ± 1.70	0.59
Young’s elastic modulus × 10^3^, kPa	3.28 ± 2.07	3.04 ± 1.74	3.29 ± 1.70	0.44
Beta-stiffness index	8.83 ± 4.56	8.42 ± 3.80	8.62 ± 3.67	0.75
Intima–media thickness, mm	0.73 ± 0.14	0.75 ± 0.14	0.73 ± 0.10	0.15
Cross-sectional wall area, mm^2^	15.9 ± 5.38	16.8 ± 4.35	15.9 ± 4.34	0.09
Lumen diameter maximum, mm	6.34 ± 0.94	6.46 ± 0.83	6.50 ± 0.80	0.48
Lumen diameter minimum, mm	5.89 ± 0.94	6.06 ± 0.83	6.12 ± 0.80	0.17

Data are arithmetic means ± SD. Data adjusted for age, gender, waist circumference, γ-glutamyl transferase, tobacco use and central systolic blood pressure.

**Table 5:** Anti-hypertensive medication use in the hypertensive group

*Type of anti-hypertensive medication*	*Hypertensive participants using medication (n = 124)*
Unspecified, n, total, (%)	61/124 (49.2)
Beta-blockers, n, total, (%)	12/124 (9.68)
Anti-adrenergics, n, total, (%)	2/124 (1.61)
Calcium channel blockers, n, total, (%)	30/124 (24.2)
Class 2 ACE inhibitors, n, total, (%)	49/124 (39.5)
Diuretics, n, total, (%)	54/124 (43.5)

n, number of participants. Unspecified: only indicated as high blood pressure pills or hypertension medication.

Table 6 reports the forward stepwise multiple regression analyses performed in the normotensive and hypertensive groups with either CD or IMT as dependent variables. As expected, CD associated with cSBP in both groups (p < 0.001), however, IMT associated independently with cSBP in the hypertensive group only (p = 0.016).

**Table 6:** Forward stepwise multiple regression analyses with carotid distensibility and carotid intima–media thickness as dependent variables

**	*Normotensive (n = 241) β (95% CI)*	*p-value*	*Hypertensive (n = 351) β (95% CI)*	*p-value*
*Distensibility (1/kPa)*				
Adjusted R^2^	0.27		0.37	
Locality (urban)	–0.16 (–0.31– –0.01)	0.031		
Age, years	–0.14 (–0.29–0.003)	0.058	–0.20 (–0.35– –0.05)	0.009
Waist circumference, cm			0.22 (0.06–0.39)	0.007
Central SBP, mm Hg	–0.44 (–0.59– –0.29)	< 0.001	–0.56 (–0.68– –0.45)	< 0.001
Heart rate, bpm	–0.10 (–0.25–0.04)	0.16	–0.12 (–0.23– –0.01)	0.030
LDL-C, mmol/l			0.08 (–0.03–0.19)	0.18
HbA_1c_ (%)			–0.14 (–0.26– –0.02)	0.018
CrCl, ml/min			–0.10 (–0.28–0.07)	0.24
ICAM-1, pg/ml	0.11 (–0.04–0.25)	0.16		
Tobacco use (no/yes)	–0.14 (–0.28–0.003)	0.058		
IMT (mm)				
Adjusted R^2^	0.25		0.35	
Locality (urban)			–0.10 (–0.20– –0.01)	0.030
Gender (male)	0.26 (0.13–0.39)	< 0.001	0.25 (0.15–0.36)	< 0.001
Age, years	0.33 (0.21–0.46)	< 0.001	0.44 (0.34–0.54)	< 0.001
Waist circumference, cm	0.14 (0.01–0.28)	0.033	0.033 0.08 (–0.02–0.19)	0.13
Central SBP, mm Hg			0.12 (0.02–0.22)	0.016
Heart rate, bpm	–0.08 (–0.20–0.05)	0.21		
LDL-C, mmol/l	0.07 (-0.05–0.19)	0.29	0.15 (0.05–0.25)	0.005
HbA_1c_ (%)			0.07 (–0.03–0.18)	0.17
C-reactive protein, pg/ml	0.16 (0.03–0.30)	0.017	0.06 (–0.04–0.16)	0.27
ICAM-1, pg/ml	0.12 (–0.01–0.24)	0.079		
γ-glutamyl transferase, U/l			–0.08 (–0.18–0.02)	0.12
Tobacco use (no/yes)			–0.08 (–0.18–0.02)	0.11
Anti-hypertension medications (no/yes)			–0.10 (–0.19– –0.003)	0.044

Data expressed as beta-values and 95% confidence intervals, p-values obtained with forward stepwise multiple regression analyses. Included in each model: locality, age, gender, waist circumference, heart rate, cSBP, LDL-C, HbA1c, C-reactive protein, ICAM-1, creatinine clearance, γ-glutamyl transferase, tobacco and anti-hypertensive medication use.

IMT, carotid–intima media thickness; CSWA, cross-sectional wall area; Max LD, maximum lumen diameter; SBP, systolic blood pressure; LDL-C, low-density lipoprotein cholesterol; HbA1c, glycated haemoglobin; ICAM-1, intercellular adhesion molecule-1.

Table 7 reports the forward stepwise regression analyses performed in the normotensive and hypertensive groups with either CSWA or maximum LD as the dependent variables. CSWA associated with cSBP (p < 0.001) in the hypertensive group only, whereas maximum LD associated with cSBP in both the normotensive and hypertensive groups.

**Table 7:** Forward stepwise multiple regression analyses with CSWA and max LD as dependent variables

**	*Normotensives (n = 241) β (95% CI)*	*p-value*	*Hypertensives (n = 351) β (95% CI)*	*p-value*
CSWA (mm^2^)				
Adjusted R^2^	0.23		0.32	
Locality (urban)			–0.11 (–0.21– –0.01)	0.022
Gender (male)	0.29 (0.15–0.42)	< 0.001	0.26 (0.15–0.37)	< 0.001
Age, years	0.29 (0.17–0.42)	< 0.001	0.43 (0.30–0.57)	< 0.001
Waist circumference, cm	0.18 (0.05–0.31)	0.008	0.02 (–0.12–0.17)	0.77
Central SBP, mmHg			0.18 (0.08–0.28)	< 0.001
LDL-C, mmol/l			0.12 (0.01–0.23)	0.026
HbA^1c^ (%)			0.08 (–0.02–0.19)	0.13
Creatinine clearance, ml/min			0.11 (–0.05–0.26)	0.18
C-reactive protein, pg/ml	0.16 (0.02–0.29)	0.025	0.09 (–0.01–0.20)	0.090
γ-glutamyl transferase, U/l	0.11 (–0.02–0.24)	0.10	–0.08 (–0.18–0.02)	0.11
Anti-hypertension medication (yes)			–0.12 (–0.22– –0.02)	0.013
Max LD (mm)				
Adjusted R^2^	0.27		0.10	
Locality (urban)			–0.08 (–0.21–0.05)	0.21
Gender (male)	0.26 (0.10–0.42)	0.001	0.22 (0.08–0.36)	0.002
Age, years	0.13 (–0.03–0.29)	0.12		
Waist circumference, cm			0.14 (-0.005–0.28)	0.061
Central SBP, mmHg	0.20 (0.05–0.35)	0.010	0.14 (0.007–0.27)	0.039
Heart rate, bpm	–0.09 (–0.24–0.06)	0.22		
LDL-C, mmol/l	–0.16 (–0.30– –0.02)	0.027	–0.14 (–0.2– –0.003)	0.045
Creatinine clearance, ml/min	0.21 (0.05–0.37)	0.011		
C-reactive protein, pg/ml			0.09 (–0.05–0.23)	0.23
ICAM-1, pg/ml	0.16 (0.01–0.30)	0.037	0.08 (–0.05–0.21)	0.25
γ-glutamyl transferase, U/l	0.17 (0.03–0.32)	0.023		

Data expressed as beta-values and 95% confidence intervals, p-values obtained with forward stepwise multiple regression analyses.

Included in each model: locality, age, gender, WC, HR, cSBP, LDL-C, HbA1c, C-reactive protein, ICAM-1, creatinine clearance, γ-glutamyl transferase, tobacco and anti-hypertensive medication use.

CSWA, cross-sectional wall area; Max LD, maximum lumen diameter; SBP, systolic blood pressure; LDL-C, low-density lipoprotein cholesterol; HbA1c, glycated haemoglobin; ICAM-1,intracellular adhesion molecule-1.

## Discussion

As expected, we found that hypertensive black Africans presented with reduced carotid distensibility when compared to normotensives. In fact, we found significant differences for all carotid wall thickness and distensibility measurements between the hypertensives and normotensives prior to adjustments. However, upon adjustment for cSBP, all differences disappeared. The direct physical measures, such as IMT, CSWA and LD, were similar between the hypertensive and normotensive groups after adjustments for both cSBP and MAP. This similarity suggests that the decreased carotid distensibility and increased carotid cross-sectional wall area of five-year sustained hypertensive Africans are, besides structural changes due to arterial degeneration, also dependent on the distending pressure, and that functional changes in the carotid artery may be more prominent than structural changes in this population.

Our findings are consistent with evidence in white populations that show increased stiffness to be due to the increased distending pressure that accompanies hypertension, suggesting a functional adaptation, and not only structural alterations of the arterial wall.^25^,^26^ In contrast to these and our findings, one study found that the acute reduction in blood pressure by nitroglycerin does not normalise large artery stiffness in essential hypertensives.^27^

It is expected that sustained high blood pressure, as seen in hypertension, would cause vascular damage by, for instance, altering the collagen–elastin ratio of the arterial wall in favour of collagen.^28^,^29^ Indeed, after adjustment for mean arterial pressure, the difference in carotid distensibility between the hypertensives and normotensives remained, therefore suggesting the presence of structural alterations. Nevertheless, in light of the significant cardiovascular burden that black populations carry,^10^,^11^ our results suggest that treatment that effectively lowers central pressure may also significantly lower the risk for stroke and other cardiovascular events.

Our results were similar for treated and untreated hypertensives; therefore treatment seems to be largely ineffective in this population. Indeed, the treatment and control of hypertension in low-income countries are largely inadequate despite half of those sampled being aware of their condition.^14^,^30^ South Africa has one of the highest hypertension rates (78%) for people over 50 years of age, but only 38% are aware of their hypertensive status and only 7.8% of those treated for hypertension have controlled hypertension.^14^ It therefore remains to be seen whether effective anti-hypertensive treatment in black Africans will result in improved carotid distensibility.

Surprisingly, the IMT was similar between the two groups after adjustments, suggesting a lack of visible structural changes in the hypertensive blacks. IMT is an important marker of the atherosclerotic burden of the carotid artery,^31^ but it may also indicate non-atherosclerotic compensatory remodelling of the arterial wall in response to hypertension.^32^ However, neither of these possibilities seems to be the case in this black population. On the other hand, IMT was independently associated with cSBP in the hypertensive group only, therefore suggesting that the continued high pulsatile load of uncontrolled hypertension may eventually mediate structural changes in the carotid artery. This result shows a similar trend to the findings of Wang et al.,^33^ confirming the relevance of central blood pressure to IMT.

We observed no differences in the inflammatory and endothelial activation markers, lipid levels and glycaemic status between the normotensives and hypertensives. Africans are generally not prone to atherosclerosis and coronary heart disease,^34^ and exhibit a favourable lipid profile,^35^,^36^ possibly explaining the similar lipid levels between the two groups. However, our results confirm the commonly found association between IMT and LDL-C level,^37^,^38^ and an association between CD and HbA_1c_ level in the hypertensive group only.

Although we did not observe structural differences after adjustment for cSBP, these results suggest glucose metabolism and lipid abnormalities may play a role in the arterial changes, although these are not yet detectable with ultrasound. Inflammation and endothelial activation (as indicated by the adhesion molecules) may not play a major role in the mediation of central arterial stiffness at this stage of disease progression. These results are unexpected in the light of previous findings, which indicate that acute and chronic inflammation are associated with stiffness of the large arteries,^39^ and that endothelial activation may be an important mediator of hypertensive vascular injury.^40^

The findings of this study should be interpreted in the context of its limitations and strengths. Our study population consisted of individuals from specific urban and rural areas in the North West Province of South Africa, and may not be representative of the whole population. We were not able to use echo-tracking techniques to determine local arterial stiffness in our field study; however, the procedures of ultrasound assessment are standardised^41^ and were performed by a single reader in a large study population. Carotid distensibility was calculated with a formula that includes cSBP, and we adjusted for cSBP. However, neither direct measurements such as IMT nor indirect variables such as carotid distensibility differed after adjustments for cSBP. Due to the cross-sectional study design, causality cannot be inferred. Although the results were consistent after several adjustments, we cannot exclude residual confounding.

## Conclusion

Although differences existed in terms of carotid structure and function between the normotensive and hypertensive Africans, it seemed to be partially accounted for by the increased distending pressure of the hypertensive group. Despite their hypertensive status, structural adaptations, such as IMT thickening, were not detectable in this African population after adjustment for potential confounders, and even before cSBP or MAP were taken into account. The classic cardiometabolic risk factors, markers of inflammation, endothelial activation and health behaviour seemed to play only a minor role in the mediation of carotid distensibility in this population at this stage of disease development. These results suggest that interventional strategies and the use of medication targeted at effectively lowering blood pressure may also lower the risk for adverse cardiovascular events in black South Africans.
